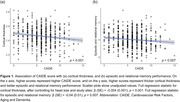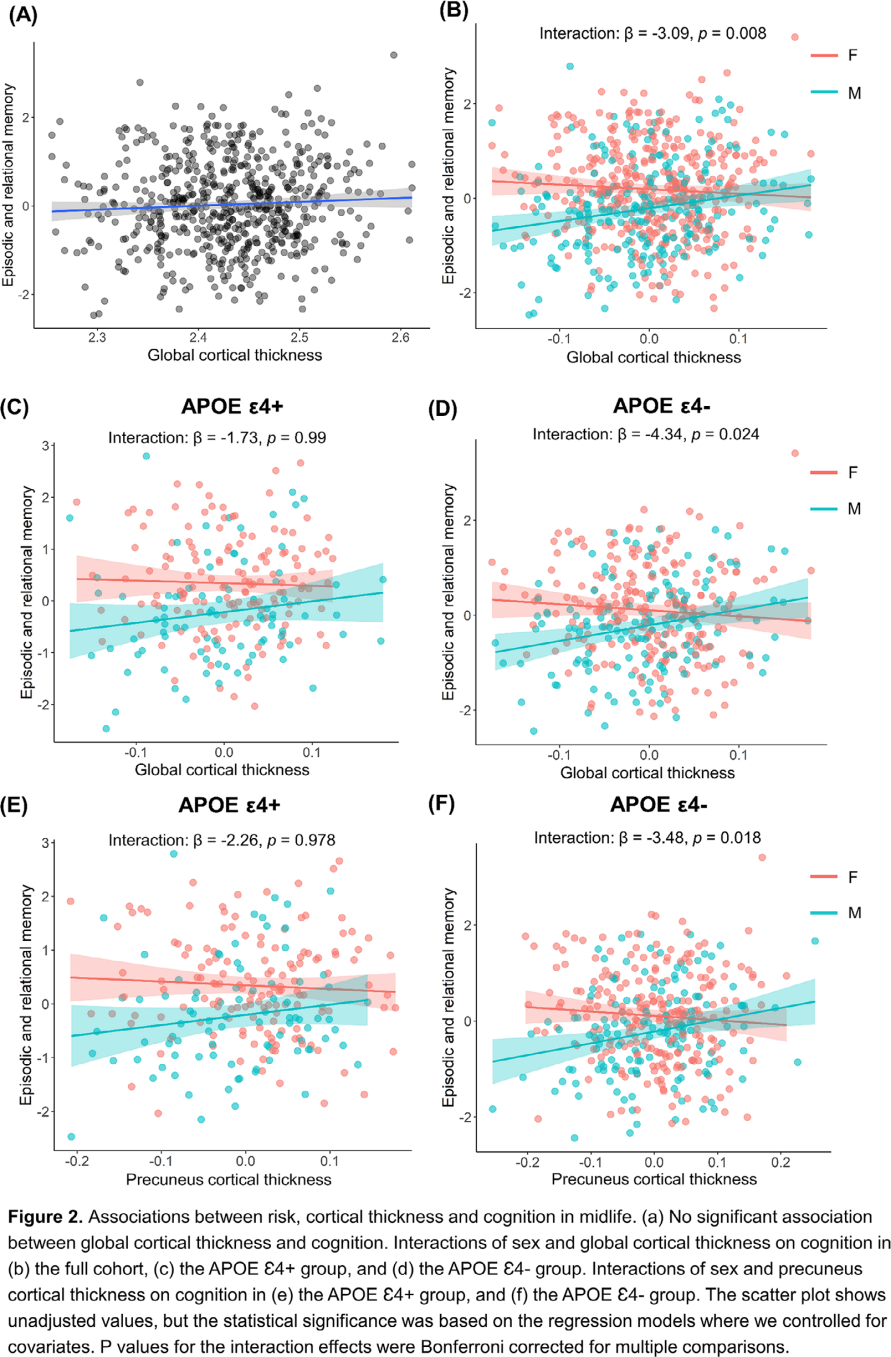# Sex differences in the relationship between cognition and brain structure in midlife individuals at risk for future dementia

**DOI:** 10.1002/alz.090268

**Published:** 2025-01-09

**Authors:** Qing Qi, Feng Deng, Maria‐Eleni Dounavi, Graciela Muniz‐Terrera, Ivan Koychev, Paresh Malhotra, John T O'Brien, Craig W Ritchie, Brian Lawlor, Lorina Naci

**Affiliations:** ^1^ Global Brain Health Institute, Trinity College Dublin, Dublin Ireland; ^2^ Trinity College Institute of Neuroscience, School of Psychology, Trinity College Dublin, Dublin Ireland; ^3^ Department of Psychiatry, University of Cambridge, Cambridge UK; ^4^ Edinburgh Dementia Prevention, University of Edinburgh, Edinburgh UK; ^5^ Department of Social Medicine, Ohio University, Athens, OH USA; ^6^ University of Oxford, Oxford, Oxon UK; ^7^ UK Dementia Research Institute Centre for Care Research and Technology, London UK; ^8^ Department of Brain Sciences, Imperial College London, London UK; ^9^ Scottish Brain Sciences, Edinburgh UK

## Abstract

**Background:**

Two‐thirds of Alzheimer’s Disease (AD) cases occur in women. Compared to men, women exhibit more rapid cognitive decline and brain atrophy in the presence of AD‐related neuropathology (Gamache et al., 2020). It is now acknowledged that AD processes are present decades before the onset of clinical symptoms (Jack et al., 2013). However, whether there are sex differences in cognition‐brain structure coupling and how AD risk affects their relationships in midlife remain unclear. In this study, we investigated associations between sex, AD risk, brain structure and cognition.

**Method:**

Structural Magnetic Resonance Imaging and detailed neuropsychological assessments were obtained for 614 cognitively healthy individuals (40‐59 years, 233 M/ 381 F) from the PREVENT‐Dementia study. Dementia risk factors were assessed by Apolipoprotein E [APOE] ε4 allele status, and the Cardiovascular Risk Factors Aging and Dementia (CAIDE) score. Multiple linear regression model was used to investigate the associations between sex, AD risk, brain structure and cognition.

**Result:**

CAIDE was negatively associated with global cortical thickness, and negatively associated with episodic and relational memory (Figure 1). We didn’t find a significant association between global cortical thickness and cognition (Figure 2a) but found a sex‐specific coupling of global cortical thickness and episodic and relational memory (Figure 2b). Such sex‐specific coupling between global cortical thickness and cognition was absent in APOE ε4 carriers, and only shown in APOE ε4 non‐carriers (Figure 2c, 2d). Furthermore, among the selected AD signature regions, cognition was decoupled from the precuneus cortical thickness in APOE ε4 carriers, and only coupled with cortical thickness of precuneus in APOE ε4 non‐carriers (Figure 2e, 2f).

**Conclusion:**

We found inherent sex‐specific differences in the coupling between brain structure and cognition. Our results suggest that these sex‐specific differences are being eroded by APOE ε4 carriership in mid‐life. Longitudinal follow‐up in this cohort will shed light on the long‐term sex‐specific impact of APOE genotype on brain structure and cognition in preclinical populations with risk for AD.